# Forging a link between mentoring and collaboration: a new training model for implementation science

**DOI:** 10.1186/s13012-016-0499-y

**Published:** 2016-10-13

**Authors:** Douglas A. Luke, Ana A. Baumann, Bobbi J. Carothers, John Landsverk, Enola K. Proctor

**Affiliations:** 1 George Warren Brown School of Social Work, Washington University in St. Louis, Campus Box 1196, One Brookings Drive, St. Louis, MO 63130 USA; 2 Oregon Social Learning Center, 10 Shelton McMurphey Blvd., Eugene, OR 97401 USA

**Keywords:** Mentoring, Scientific collaboration, Training, Network analysis, Implementation science, Team science, Implementation Research Institute

## Abstract

**Background:**

Training investigators for the rapidly developing field of implementation science requires both mentoring and scientific collaboration. Using social network descriptive analyses, visualization, and modeling, this paper presents results of an evaluation of the mentoring and collaborations fostered over time through the National Institute of Mental Health (NIMH) supported by Implementation Research Institute (IRI).

**Methods:**

Data were comprised of IRI participant self-reported collaborations and mentoring relationships, measured in three annual surveys from 2012 to 2014. Network descriptive statistics, visualizations, and network statistical modeling were conducted to examine patterns of mentoring and collaboration among IRI participants and to model the relationship between mentoring and subsequent collaboration.

**Results:**

Findings suggest that IRI is successful in forming mentoring relationships among its participants, and that these mentoring relationships are related to future scientific collaborations. Exponential random graph network models demonstrated that mentoring received in 2012 was positively and significantly related to the likelihood of having a scientific collaboration 2 years later in 2014 (*p* = 0.001). More specifically, mentoring was significantly related to future collaborations focusing on new research (*p* = 0.009), grant submissions (*p* = 0.003), and publications (*p* = 0.017). Predictions based on the network model suggest that for every additional mentoring relationships established in 2012, the likelihood of a scientific collaboration 2 years later is increased by almost 7 %.

**Conclusions:**

These results support the importance of mentoring in implementation science specifically and team science more generally. Mentoring relationships were established quickly and early by the IRI core faculty. IRI fellows reported increasing scientific collaboration of all types over time, including starting new research, submitting new grants, presenting research results, and publishing peer-reviewed papers. Statistical network models demonstrated that mentoring was strongly and significantly related to subsequent scientific collaboration, which supported a core design principle of the IRI. Future work should establish the link between mentoring and scientific productivity. These results may be of interest to team science, as they suggest the importance of mentoring for future team collaborations, as well as illustrate the utility of network analysis for studying team characteristics and activities.

**Electronic supplementary material:**

The online version of this article (doi:10.1186/s13012-016-0499-y) contains supplementary material, which is available to authorized users.

## Background

Americans with mental disorders receive sub-optimal care due to formidable challenges in the implementation, sustainability, and scale-up of evidence-based treatments [1, 2]. Dissemination and implementation of research findings into practice are necessary to close the gap between what is known to be an effective treatment and what is currently being implemented in usual care [3]. Given persistent quality gaps, the NIH encourages research (e.g., “Dissemination and Implementation Research in Health,” [4]) on strategies to improve the adoption, implementation, and sustainment of evidence-based interventions in usual care. Yet, that research cannot accrue without a cadre of well-trained investigators.

### Mentoring, collaboration, and implementation science

Training investigators for the rapidly developing field of implementation science requires both mentoring and collaboration. The National Institute for Mental Health (NIMH) has prioritized team science and mentoring in all efforts to prepare the research workforce. A 2008 NIMH council workgroup report, “*Investing in the Future*,” proposed a “phenotype” of the NIMH researcher of the future: transdisciplinary scientists, team players in a collaborative scientific enterprise, and translators [5]. The report proposed that a body of researchers with these three “T’s” capture the research *phenotypes* needed to carry out the type of research needed to reduce the research-practice gap in mental health; it emphasized that “mentoring is essential” and called for “national mentoring networks,” to achieve NIMH objectives [5]. Mentoring has been shown to contribute to research productivity and career success [6, 7]. Burnham and colleagues [8] outline mentor qualities that facilitate career development, including resources/ideas, editorial support with prompt feedback, and positive encouragement. The literature on mentoring in the health sciences informs our approach, particularly on interdisciplinary training. In summary, we conceptualize mentoring as an interactive process aimed at promoting learning and development of the trainee [9, 10].

Responding to the 2008 report, the National Institutes of Health (NIH) supported training programs in dissemination and implementation (D&I) research by placing heavy emphasis on mentoring, both from faculty to training participants and between trainees themselves. Since 2009, at least three training programs focused in implementation science have been pairing trainees with mentors with the goals of expanding the D&I research community and advancing the intellectual capital of this still developing field in the USA. The Implementation Research Institute was the first training institute focused on mental health implementation science [11]. It was followed by the Training in Dissemination and Implementation Research in Health (TIDIRH) [12] and the Mentored Training in Dissemination and Implementation Research in Cancer (MT-DIRC) research institute [13]. The focus of this evaluation study is to examine how mentoring was associated with scientific collaboration in the IRI training institute.

### Team science and D&I research

An interest in team science is increasing in many areas of science, but scientific collaboration and cross-disciplinary partnerships are of particular interest to D&I research [14, 15]. (In this paper, we follow the basic definitions of inter- and transdisciplinary science [16]. Notably, specific scientific collaborations are characterized as inter- or multidisciplinary, while the science that arises from these collaborations may be properly viewed as transdisciplinary). D&I research itself is closely related to and draws often from such fields as health services research, intervention development and testing, improvement science, human factors engineering, and organizational research [11, 12]. D&I research draws from these and other fields for both theory and research methods, and the field’s distinct or unique theories and methods are inherently multidisciplinary, if not transdisciplinary.

The science of team science has emerged as an approach to evaluate the processes and outcomes of partnered research [17, 18]. Social network analysis (SNA) is uniquely suited to study collaborative relationships [19]. SNA has been used to study cross-disciplinary collaborations in the National Institutes of Health’s Clinical and Translational Science Awards (CTSA) programs [20], as well as other large-scale research initiatives such as the Transdisciplinary Tobacco Use Research Centers (TTURCs) and NCI’s Physical Sciences—Oncology Centers (PS-OC) [21, 22].

### The implementation Research Institute (IRI)

The IRI is a 2-year training institute in mental health implementation science, supported by a National Institute of Mental Health R25 grant and the Department of Veterans Affairs. During the first round of (5-year) funding, 43 fellows in four cohorts were trained at IRI. Fellows attended two annual weeklong trainings at Washington University in St. Louis, traveled for a site visit on still-in-the-field implementation projects, attended implementation science conferences, and received research pilot funding. Further description of IRI can be found elsewhere [11].

The fundamental design of IRI was to provide strong mentorship in D&I science to support new scientific collaborations, with an overall goal of stimulating IRI fellows’ production of D&I scholarly products such as papers, presentations at conferences, and grants. Thus, the program was designed as a learning collaborative in implementation science, for both fellows and faculty members [23]. Based on this learning collaborative approach, even though we anticipated that mentoring would start with guided interactions between experienced faculty and the IRI fellows (see below), we intended and conveyed the expectation that mentoring and collaboration relationships would arise over time among all IRI participants (faculty and fellows alike).

Mentoring at IRI was done by matching each fellow with one of the core faculty members. One innovative aspect of IRI, compared to the other training methods in D&I at the time, was the monthly mentoring via phone or video conference calls for a 2-year period by core faculty to IRI fellows. The requirement that each fellow also have a mentor at his or her home institution focused on grant writing and career development enabled the IRI core faculty mentors to focus specifically on the fellow’s development of research grants in implementation science.

The central component in the training—the 5-day summer institute—was shaped to foster networking and collaboration. We required in-residence immersion for 5 days and nights and used small group sessions for faculty-to-fellow and fellow-to-fellow feedbacks on the scholarly products that fellows worked to advance during the week. We fostered informal networking by providing dinners in and off site, and convening IRI gatherings during the year, specifically at annual D&I conferences [11]. While fellows were not explicitly taught to collaborate, the institute structure—particularly the face-to-face training and site visit—facilitated networking and collaboration. We anticipated that the collaborative network emergent during the institute would provide a basis for future collaboration and ongoing consultative relationships.

The design of our training was also guided by a team science approach [11]. The grant proposal to secure IRI funding stated a pedagogical philosophy that interpersonal activity, specifically intellectual exchanges between fellows and senior scholars, is key to the science-building process [24]. Our faculty were drawn from various fields (including social work, psychology, public health, epidemiology, and sociology); and our fellows were from six different disciplines [11]. This breadth facilitated the promotion of multidisciplinary collaboration and the establishment of team science.

This paper presents findings on the link between mentoring and new collaborations in implementation science, employing social network analysis. Social network analysis is suited to examine the development of collaboration in the IRI, given the centrality of mentoring and team science to NIMH-supported research training. This evaluation study seeks to answer the extent to which strong mentoring relationships are associated with increased scientific collaboration. Specifically, the goals of this paper are to

1. Describe and map the *mentoring networks* across three IRI cohorts;
2. Similarly describe and map the *collaboration relationships* among the IRI network (fellows and faculty) as they develop during the time of the IRI funding; and
3. Explore and model the relationship between mentoring and subsequent scientific collaborations.

## Methods

This is an evaluation study that examines the dynamics of mentoring and collaboration among participants of the IRI from 2012 to 2014. The study uses network data collected from all IRI participants during each summer in-person training workshop. Three types of network ties were collected: frequency of contact, receiving or providing mentoring, and type of scientific collaboration.

### Participants

The IRI participants played one of three roles. *Fellows* (43 across four cohorts) were scholars who were selected through a competitive national application process and attended two annual training workshops. *Core faculty* were the core set of D&I senior scholars who directed and managed the IRI workshops and provided the most active and direct mentoring to the fellows. The institute had seven core faculty for the first 2 years (2012 and 2013); core faculty were increased to nine in 2014 with support from a National Institute on Drug Abuse (NIDA) supplement. *Expert faculty* were a larger set of researchers who had particular expertise relevant for IRI training. The set of core faculty remained mostly constant throughout the lifetime of the IRI initiative (seven of nine core faculty participated in all three waves, the other two attended during the final two waves), while expert faculty generally attended only one of the annual workshops. Core faculty stayed in residence during each summer institute, but expert faculty members were invited to participate for 1 or 2 days and to be in residence for only one night [11].

### Data collection

An IRI participant survey was developed to collect network information from all IRI participants each year. The survey was introduced and described to the attending IRI members at each summer institute. Both current and former institute participants were invited to participate each year. The survey was administered with Qualtrics, an online web-based survey platform [25].

### Measures

The three network items were based on items that have been used successfully in past network studies [26] with slight modifications to accommodate IRI activities. *Contact:* “Please indicate which of the following people you know and how frequency you are in contact with them on the list below: (1) I don’t know this person, (2) I know this person but we have no contact, (3) I am in contact with this person yearly, (4) I am in contact with this person monthly, (5) I am in contact with this person weekly,” followed by a list of the names of all individuals who had participated in IRI so far. Only the names of the individuals participants indicated being in contact with at least yearly appeared in the next two questions. *Mentoring:* “On the list below please indicate which individuals have either mentored you or been mentored by you in the past year: (1) I mentored them, (2) They mentored me, (3) Neither.” *Collaboration:* “Please check any of the collaboration activities you have engaged in with the following individuals during the past year: (1) Worked together on developing new research, (2) Submitted a grant, (3) Presented research results, (4) Published a paper, (5) None of these.” Working together on developing new research was designed to capture the early stages of research collaboration, before formal grants are submitted or results are disseminated. Member pairs were considered linked if one or both indicated a relationship, following established social network data management protocols [27]. Thus, links to members who did not participate in the survey were constructed when indicated by participating members.

In addition to the three network items, participants were asked to indicate what scientific discipline best described their scholarly work and expertise, based on the NIH Field of Training list [28]. From an initial list of 205 categories, participants were classified into one of three broad disciplines: allied health (social work, public health, nursing, dentistry, etc.), clinical/medicine (clinical psychology, psychiatry, geriatrics, etc.), and social science/statistics/methodology (non-clinical psychology, informatics, statistics, etc.).

### Data analysis

The network data were analyzed using three types of analytic approaches: visualization, descriptive statistics, and statistical modeling. Exponential random graph models (ERGMs) were used to build and test predictive models of network ties among the IRI fellows and core faculty [29]. All network analyses were conducted using the *statnet* (version 2014.2.0), *igraph* (0.7.1), and *ergm* (3.24) packages in R (Additional files 1, 2, 3, 4, and 5).

## Results

A total of 94 people participated in IRI from 2012 to 2014, including 9 core faculty, 42 expert faculty, and 43 fellows. Response rates for the surveys were 80 % (2012), 76 % (2013), and 80 % (2014). Basic descriptive summaries of mentoring and collaboration suggest that the IRI design was working effectively. One hundred percent of IRI fellows reported receiving some type of mentoring each year, with the average number of received mentoring relationships ranging from 5.8 (in 2013) to 6.5 (2012). Similarly, all fellows reported some type of collaboration with others (fellows, core faculty, and expert faculty) for each of the 3 years. Average number of collaborations ranged from 6.5 in 2012 to 8.0 in 2014.

### General IRI mentoring and collaboration network characteristics

**Fig. 1:**
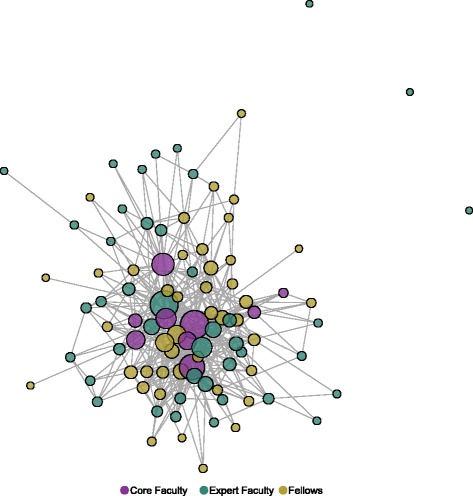
Total collaboration network among all IRI members, 2014

Figure 1 presents the network of all collaboration ties (new research, grant submission, presenting results, and publishing) for all 94 members of IRI in 2014, at the end of the third wave of data collection. The nodes are color coded by role (i.e., fellows, core faculty, and expert faculty), and a tie connects two nodes if one or both of those two IRI members reported any type of scientific collaboration in the previous year. The sizes of the nodes correspond to the overall degree, that is, the number of collaboration partners. This figure illustrates the dense, interconnected nature of the IRI participant network and the high level of scientific collaboration that was occurring near the end of the IRI initiative. Moreover, the structure of the network suggests that the training and mentoring model of IRI is operating as planned. In particular, the core faculty members (purple nodes) are more prominent in the network compared to the expert faculty, as demonstrated by their central placement in the network layout and larger node sizes. Conversely, the only isolates (unconnected nodes) among the IRI collaboration network are expert faculty, who were not designed to have intense, ongoing collaborations with IRI fellows.

Table 1 presents detailed network characteristics for the three IRI networks (contact, mentoring, and collaboration) and how they changed over the three waves (2012–2014). Not surprisingly, the contact network shows the highest density (proportion of observed to total possible ties) and average degree (number of direct ties for a particular node). Density of collaboration ties is higher than mentoring across all 3 years. Betweenness centrality is highest for the collaboration ties, suggesting that there are a small number of prominent scholars that are active researchers, and their collaboration ties connect to different parts of the IRI network. Modularity is a measure of the extent to which the tie patterns in a network can be explained by distinct subgroups or communities in the network. Here, role modularity is assessing whether the observed ties tend to exist within the three role categories (core faculty, fellows, expert faculty) or across these categories. Modularity scores can range from −0.5 to +1, higher scores indicating more within-group ties relative to across group ties. The negative role modularity scores for mentoring suggest that mentoring is, in fact, occurring across the role categories, as it should be for the IRI training program. Initial exploration of the pattern of collaboration ties within and across the three broad disciplinary categories revealed that there was a slight tendency to report collaborations *across* disciplines relative to *within* disciplines (53 %). This interdisciplinary tendency increased slightly in subsequent waves (59 % in 2013; 57 % in 2014). (Detailed results not reported here but are available from the authors.)

**Table 1:** Network characteristics of IRI participants from 2012 to 2014

	2012					2013					2014				
Name	Size	Density	Average degree	Betweenness centrality	Role modularity	Size	Density	Average degree	Betweenness centrality	Role modularity	Size	Density	Average degree	Betweenness centrality	Role modularity
Contact—full^a^	65	0.41	26.25	0.08	0.03	85	0.27	22.71	0.13	0.02	94	0.25	23.55	0.11	0.01
Mentoring—full	65	0.06	7.29	0.10	−0.17	85	0.05	7.62	0.13	−0.12	94	0.04	7.19	0.05	−0.19
Collaboration—full	65	0.11	7.02	0.26	0.03	85	0.09	7.76	0.16	0.04	94	0.10	9.49	0.17	0.03
Contact—reduced^b^	38	0.67	24.74	0.02	−0.04	50	0.39	19.20	0.13	−0.05	52	0.39	19.65	0.11	−0.08
Mentoring—reduced	38	0.10	7.47	0.09	−0.27	50	0.07	6.68	0.03	−0.18	52	0.07	6.85	0.01	−0.28
Collaboration—reduced	38	0.16	5.89	0.17	−0.01	50	0.12	5.92	0.30	−0.00	52	0.14	6.96	0.24	−0.00

^a^Full networks include core faculty, fellows and expert faculty

^b^Reduced networks include only core faculty and fellows

**Fig. 2:**
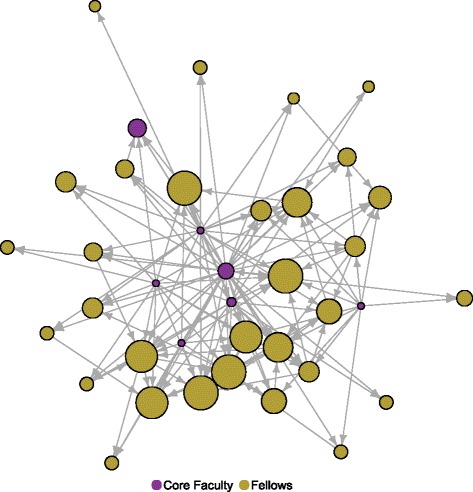
Mentoring network among IRI fellows and core faculty, 2012

Figure 2 illustrates more clearly the nature of mentoring relationships as observed at the first wave of data collection in 2012. Here, the directed ties show the reported mentoring received by each IRI participant (core faculty and fellows) and the nodes are sized by the number of incoming mentoring relationships. This figure also shows that IRI was working as designed with more mentoring relationships being observed going from core faculty to fellows. (In 2012, there were only seven core faculty.) Although the IRI networks were larger in 2013 and 2014 (made up of more than one cohort), the amount of mentoring received remains fairly stable; the average degree of mentoring relationships ranges from 6.68 to 7.47 (Table 1).

**Fig. 3:**
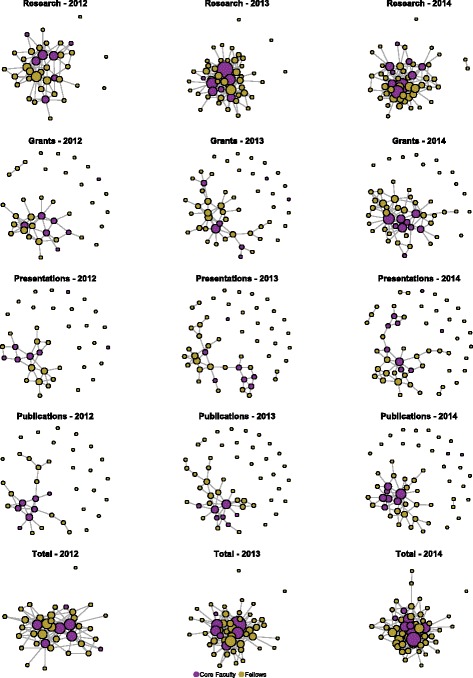
Collaboration networks by type and by year, 2012–2014

Figure 1 and Table 1 present information on overall collaboration, regardless of type. In Fig. 3 and Table 2, collaboration relationships are broken down by four basic types: new research, new grant submissions, scientific presentations, and new paper publications. Table 2 shows that over time, all four types of scientific collaborations increased among core faculty and fellows, while Fig. 3 shows that collaborations occur across all four types. New research collaborations were the most frequently occurring type of collaboration. Of particular note is that in 2014, IRI participants reported 82 new grant collaborations. The breakdown of this number is interesting; 35 of these new grant submission collaborations were between core faculty and fellows, while 29 of them were among just fellows (meaning the collaboration is occurring between two different IRI fellows). Only 18 of these were among the core faculty. So, 78 % of the reported new grant collaborations involved the IRI fellows. The bottom of Table 2 shows how many different types of collaborations were reported by IRI members for each year. Scientific collaboration among IRI members was often multiplex, being made up of multiple types of collaboration. In 2012, 39 % of the IRI members reporting scientific collaboration had two or more types; in 2014, this had grown to 48 %. Given the way that these collaborations are coded, we can also say that a number of IRI members were involved with both new research planning (new research and grants) and research dissemination (presentations and publications) collaborations within the same year. For example, in 2014, 34 IRI members reported three or four types of collaboration ties.

**Table 2:** Patterns of collaboration over time among IRI core faculty and fellows (numbers are reported ties for each network)

	2012	2013	2014
Types of collaboration			
New research	89	131	142
Grants	41	49	82
Presentations	28	39	47
Publications	26	40	55
Number of different types of collaboration			
1 type of collaboration	68	84	95
2 types of collaboration	25	35	52
3 types of collaboration	12	15	20
4 types of collaboration	7	14	14

### Modeling the relationship between mentoring and subsequent scientific collaborations

One of the primary assumptions of the IRI program is that scholarly productivity of IRI fellows will be accelerated by fostering mentoring relationship built during IRI activities, and a primary mechanism by which mentoring affects productivity is via establishment of new and effective scientific collaborations. In this section, we report analytic results focusing on the relationship between mentoring and collaboration among IRI participants.

**Fig. 4:**
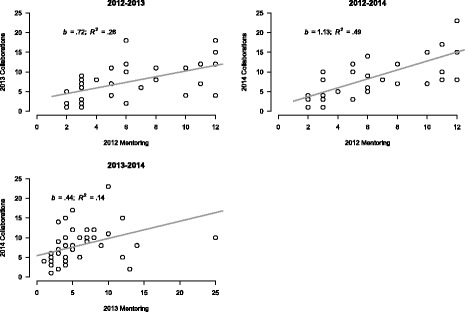
Relationship of mentoring to collaboration among IRI fellows

Simple bivariate correlations between mentoring received and reported total collaborations range from a low of 0.37 (for mentoring and collaborations reported in 2013) to a high of 0.70 for mentoring received in 2012 and collaborations reported in 2014. Figure 4 shows the strength and patterns of the lagged correlations that we observed between the amount of mentoring received by IRI participants and the number of reported scientific collaborations. The regression reported in the 2012–2014 panel in Fig. 4 indicates that for every additional mentoring relationship, there was a predicted increase of new collaborations of any sort of 1.13. Breaking this down for the specific types of scientific collaborations, for every additional mentoring relationship in 2012, we found a predicted increase of 0.90 for new research, 0.49 for new grants, 0.45 for presentations, and 0.38 for publications. This pattern also reflects the temporal nature of scientific collaboration and productivity. Planning new research has to happen prior to disseminating the fruits of that research.

These simple network and bivariate analyses are suggestive, but we can test our hypothesis of mentoring leading to collaboration more explicitly by using new stochastic network modeling techniques. Exponential random graph models are a relatively new technique that combines maximum likelihood estimation with network simulations to be able to build statistical models of networks [29]. ERGMs are particularly useful for testing hypotheses about network relations, and they have started to be applied more widely in public health [27].

Table 3 presents the results of three ERGMs predicting the presence of collaboration ties among IRI fellows and core faculty in 2014, based on a small number of network member and network structure predictors. The initial model is a null model used as a baseline comparison. It has no predictors other than an edge constant term that constrains the model to produce simulated networks that have the same size and density as the observed IRI collaboration network. The next model (model 1) adds three covariates. *Same discipline* captures the homophily effect of discipline (when both IRI participants have the same discipline) on the likelihood of observing a collaboration tie. *Role-fellow* captures the simple main effect of role, in this case being an IRI fellow. Finally, GWESP (geometrically weighted edgewise shared partners) is a network structural term that captures the patterns of transitivity in the observed network. Transitivity is the common social pattern of closure, where if one person is connected to two other people (via friendship, for example), then there is an increased probability that the two other people are also connected. Local structural covariates such as GWESP are typically included in ERGMs as they improve their stability and increase the fit of the models to the observed data [30]. Model 2 then adds two additional terms to the previous model that assess the effects of mentoring received in 2012 and 2013 on collaboration in 2014.

**Table 3:** Stochastic network model predicting any collaboration in 2014 for IRI fellows and core faculty

	Null Model			Model 1				Model 2			
	Est.	SE	*p*	Est.	OR	SE	*p*	Est	OR	SE	*p*
Edges (constant)	−1.46	0.10	0.000	−2.09	–	0.58	.000	−1.69	–	0.60	0.005
Same discipline				0.60	1.82	0.20	0.002	0.57	1.77	0.21	0.006
Role—fellow				−1.05	0.35	0.19	0.000	−1.65	0.19	0.28	0.000
GWESP (clustering)				1.07	2.92	0.24	0.000	0.86	2.36	0.24	0.000
Mentoring—2012								0.084	1.09	0.02	0.001
Mentoring—2013								−0.002	0.99	0.01	0.898
AIC	684			572				560			

The results of models 1 and 2 both show that there is a positive discipline homophily effect on collaboration; collaboration is more likely to be observed between two IRI members from the same discipline (allied health; clinical/medicine; social sciences). Also, being an IRI fellow reduces the likelihood of collaboration—this simply reflects the high level of collaboration among the small number of core faculty. The GWESP term is also significant, suggesting that there is transitivity among the IRI members. After controlling for these covariates, model 2 finds that mentoring received in 2012 has a significant and positive relationship with the likelihood of collaboration in 2014 (*p* = 0.001). In addition to interpreting the patterns and sizes of the fitted parameters, the quality of an ERGM can be assessed by examining its goodness-of-fit. Goodness-of-fit diagnostics were run to assess how closely a set of 1000 simulated networks based on model 2 match the observed IRI 2014 collaboration network on four different network characteristics: minimum geodesic distance (compactness), the edgewise shared partner distribution, the degree distribution, and the triad census (pattern of triangles). For a good fit, the value of the statistic (such as number of nodes with degree = 1) of the observed network should fall within an empirical 95 % confidence interval calculated from the simulated model-based networks. Out of 61 network statistics, 58 of them fall within the confidence bounds, suggesting that model 2 fits the data well; and that with just five predictors, our ERGM is able to produce predicted networks that look very much like the 2014 IRI collaboration network. (Detailed goodness-of-fit analytic results are available from the authors.)

The ERGM results reported in Table 3 were for a model that predicts collaboration ties among IRI participants for any type of collaboration. ERGM models were also run for each of the four specific scientific collaboration ties: new research, grants, presentations, and publications. The same model 2 structure was used for each of these four more specific collaboration ties. Table 4 presents the parameter estimate for the relationship between mentoring received in 2012 and the likelihood of a collaboration in 2014, along with the associated odds ratios, standard errors, and *p* values. These results show that mentoring received 2 years earlier is still a significant predictor of collaborations for new research, grants, and publications. In fact, the strength of the relationship is highest for grant and publication collaborations.

**Table 4:** Strength of 2012 mentoring predictor for five types of 2014 collaborations

Type of collaboration	Est.	OR	SE	*p*
New research	0.061	1.06	0.023	0.009
Grants	0.127	1.14	0.043	0.003
Presentations	0.071	1.07	0.037	0.057
Publications	0.111	1.12	0.047	0.017
Any collaboration	0.084	1.09	0.025	0.001

Parameter estimates are based on full multivariate ERGM; adjusted for discipline, role, local clustering, and 2013 mentoring

**Fig. 5:**
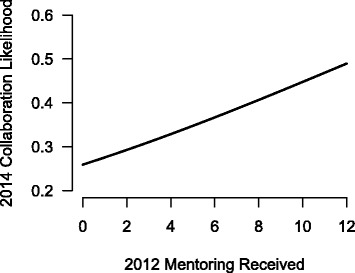
Model estimates of increased likelihood of collaboration in 2014 as a function of received mentoring 2 years earlier in 2012

The parameters in ERGMs can be treated like logistic regression parameters, because of the exponential nature of the model that is predicting the likelihood of observing a specific network tie. Therefore to understand the meaning of the parameters, the logistic transform must be used on the estimated parameters to put the predicted values into the appropriate (0, 1) probability range. Figure 5 presents the predicted probabilities of observing a collaboration tie in 2014, for different amounts of mentoring received in 2012. This forecast assumes that the dyad is made up of IRI members from the same discipline, and that one of the dyad members is a fellow, and the other a core faculty member. As can be seen, the probability of a scientific collaboration between any particular fellow and core faculty ranges from a low of 0.26 to a high of 0.49. More specifically, by increasing the number of mentoring relationships received by an IRI fellow from four to eight results in a 24 % increase in the likelihood of future collaborations (0.33 to 0.41). In other words, every additional mentoring relationship received by a fellow increases the likelihood of collaboration 2 years down the road by approximately 6 %.

## Discussion

These results demonstrate the utility of scientific training that explicitly connects mentoring by experienced implementation scientists with subsequent scientific collaboration among trainees and other scientists. First, the network analysis demonstrates that the IRI developed a tight-knit community of D&I research scholars. The program was successful in forming mentoring relationships among IRI participants, with mentoring ties strongest between core faculty and IRI fellows, consistent with program design. To our knowledge, the IRI was the first implementation science researcher training program that provided direct mentoring activities over an extended period of time (i.e., not just within the weeklong period of face-to-face contact). This novel training approach can serve as a model for other federally funded scientific training initiatives.

Second, we found evidence for a connection between mentoring and scientific collaboration of all types. The SNA results demonstrate a sizeable impact of the mentoring relationships on future scientific collaborations, with every additional mentoring relationship predicting increases in new scientific collaborations on grants, presentations, and publications. These findings support a key principle in the NIMH National Advisory Council’s Workgroup on developing the research workforce, *Investing in the Future* (2008): that mentoring is one of the elements essential for the development of a successful research career, and mentoring should be directed toward developing researchers who can work collaboratively [5]. Collaborative research was noted as particularly important for speeding translation of research into new treatments and interventions for mental disorders, and we would assert for the discovery of ways to disseminate and implement those interventions into routine settings of care. The finding that mentoring showed relationships with scientific collaboration of all types 2 years later confirms the benefit of requiring fellows to participate in the institute for 2 years with continued mentoring. A 2-year program is consistent with the realities that both relationships and scientific products require considerable time to develop, and that relationships strengthen over a period of continued interaction. Moreover, the stability of mentoring relationships from faculty to fellows demonstrated as the number of participants grew (years with both first and second year fellows) indicates that having two cohorts in residence did not reduce the quality of impact of mentoring to fellows. In fact, fellows provided significant mentoring to one another, particularly second year to first year fellows.

Studies of the outcomes of large research and training initiatives, such as the TTURCs, the Transdisciplinary Research on Energetics and Cancer centers (TREC), and the Clinical and Translational Science Awards (CTSA), have steadily moved from early descriptive and case study work to more recent attention to core aspects of team science [31, 32]. Investment in the scientific enterprise works partly by providing basic infrastructure and resources to researchers; however, scientific collaboration should also be seen as both an important outcome of such investment and an intermediary phase between research investment and scientific productivity outcomes such as grant submissions and empirical publications. One of the most important contributions of this study is to unpack the return-on-investment “black box” and identify a potential mechanism by which research investment actually leads to scientific collaboration and subsequent scientific productivity.

The study reported here has a number of notable design and analysis strengths, including longitudinal data collection, multiple overlapping cohorts, measuring scientific collaboration both generally and specifically (i.e., four types of scientific collaboration), and using statistical network modeling to establish the quantitative relationship between mentoring and collaboration. This is the first study, to our knowledge, to model the relationship between mentoring and scientific collaboration. Moreover, it reflects the utility of network analysis for studying mentoring and collaboration specifically and for enhancing D&I science more generally [33].

However, a number of design and analytic limitations should be kept in mind when interpreting these results. The mentoring and collaboration measures were based on self-report and there is the potential for some social desirability bias. In fact, when asked about mentoring relationships, we did not (a) control for context (e.g., did mentoring happened during the institute or did it occur during the 2-year phone meetings?) because the entire IRI network—alums and fellows—responded to the same survey at the same time nor (b) ask for mentoring frequency. Future studies may disentangle when and how often mentoring occurs to understand the details of the mentoring process and how it affects collaboration among members of the network. This was an observational study, so the causal relationship between mentoring and collaboration cannot be firmly established absent a control group. (Although it is not clear that a valid control group could be established for the IRI program.) More specifically, we cannot specify the extent to which collaboration ties were the result of mentoring within IRI or the result of other mentoring that occurred outside of IRI as well as other non-mentoring aspects of the program such as instruction in D&I research methods. The small number of core faculty involved in IRI across all the years raises questions of generalizability and scalability. Would the same relationships be found with a different set of faculty? Also, although we found that scientific collaborations among IRI fellows and core faculty were slightly more likely to be interdisciplinary in nature over the course of the trainings, this was not examined in detail in the statistical models that were focused on mentoring and collaboration. This will be examined in more detail in future work.

## Conclusions

The strong scientific collaboration network and high impact mentoring demonstrated in the first few years of the IRI provide an important foundation for future training. The IRI renewal, recently awarded by the NIMH, will support 5 years (2016–2021) of training deliberately designed as a mentoring collaborative. We will continue to assess the development and impact of mentoring ties, extending our analysis to examine not only collaboration but scientific productivity and other markers of career success, such as leadership to the field of D&I science.

## Additional files

Additional file 1: IRICodebook.pdf: codebook for the included data files. (PDF 380 kb)
Additional file 2: Contact[year].net: Pajek.net file for contact network for each year. (ZIP 11 kb)
Additional file 3: Collaboration[year][activity].net: Pajek.net file for collaboration network for each year and activity. (ZIP 24 kb)
Additional file 4: Mentor[year].net: Pejak.net file for mentor networks for each year. (ZIP 5 kb)
Additional file 5: Attributes[year].csv: comma-separated file of IRI member attributes for each year. (ZIP 1 kb)

## Abbreviations

CTSA: Clinical and Translational Science Award
D&I: Dissemination and implementation
ERGM: Exponential random graph models
GWESP: Geometrically weighted edgewise shared partners
IRI: Implementation Research Institute
MT-DIRC: Mentored Training in Dissemination and Implementation Research in Cancer
NIDA: National Institute on Drug Abuse
NIH: National Institute of Health
NIMH: National Institute of Mental Health
PS-OC: Physical Sciences—Oncology Centers
SNA: Social network analysis
TIDIRH: Training in Dissemination and Implementation Research in Health
TTURC: Transdisciplinary Tobacco Use Research Centers
