# Supplementary material for: Forging a link between mentoring and collaboration: a new training model for implementation science
Source: Implement Sci. 2016 Oct 13;11:137. doi: 10.1186/s13012-016-0499-y (PMC5062835; doi:10.1186/s13012-016-0499-y)
Supplement: Additional file 1: — IRICodebook.pdf: codebook for the included data files. (PDF 380 kb) [file 13012_2016_499_MOESM1_ESM.pdf]

IRI Network Data Codebook

Networks

Contact

Network	Links	Direction
Contact	Valued (average)	Non-directed

Value	Label
1	Yearly
2	Monthly
3	Weekly

Collaboration

Network	Links	Direction
NewResearch	Dichotomous	Non-directed
Grant	Dichotomous	Non-directed
PresentResearch	Dichotomous	Non-directed
PublishPaper	Dichotomous	Non-directed
Any	Valued – sum of the above	Non-directed

Mentor

Network	Links	Direction
Mentor	Dichotomous	Directed

Attributes

Variable	Label
NodeID	Vertex number in .net file
Label	Vertex label in .net file
Role	IRI member role<br>1 = Fellow<br>2 = Core Faculty<br>3 = Expert Faculty
Discipline	Broad scientific discipline<br>1 = Allied Health<br>2 = Clinical/Medicine<br>3 = Social Science/Statistics/Methodology
